# Supplementary material for: Time-series transcriptome analysis identified differentially expressed genes in broiler chicken infected with mixed Eimeria species
Source: Front Genet. 2022 Aug 8;13:886781. doi: 10.3389/fgene.2022.886781 (PMC9393255; doi:10.3389/fgene.2022.886781)
Supplement: Supplementary file 2 [file DataSheet1.ZIP › 7dpi_KEGG.Gsea.1625115961950/KEGG_JAK_STAT_SIGNALING_PATHWAY.html]

Details for gene set KEGG\_JAK\_STAT\_SIGNALING\_PATHWAY[GSEA]

|  || Dataset | TMM\_7dpi\_gct\_format\_7dpi\_gct\_format.Class\_4dpi.cls #PC\_versus\_NC.Class\_4dpi.cls #PC\_versus\_NC\_repos |
| Phenotype | Class\_4dpi.cls#PC\_versus\_NC\_repos |
| Upregulated in class | 0 |
| GeneSet | KEGG\_JAK\_STAT\_SIGNALING\_PATHWAY |
| Enrichment Score (ES) | -0.46877635 |
| Normalized Enrichment Score (NES) | -1.6666307 |
| Nominal p-value | 0.0 |
| FDR q-value | 0.07231652 |
| FWER p-Value | 0.3978 |
Table: GSEA Results Summary

  

Fig 1: Enrichment plot: KEGG\_JAK\_STAT\_SIGNALING\_PATHWAY      
 Profile of the Running ES Score & Positions of GeneSet Members on the Rank Ordered List

  

| SYMBOL | TITLE | RANK IN GENE LIST | RANK METRIC SCORE | RUNNING ES | CORE ENRICHMENT || 1 | STAT1 | na | 103 | 1.240 | 0.0134 | No |
| 2 | STAT2 | na | 285 | 0.953 | 0.0151 | No |
| 3 | IRF9 | na | 457 | 0.776 | 0.0145 | No |
| 4 | BCL2L1 | na | 568 | 0.700 | 0.0177 | No |
| 5 | SPRED1 | na | 844 | 0.575 | 0.0048 | No |
| 6 | SOCS1 | na | 864 | 0.566 | 0.0133 | No |
| 7 | IL13RA2 | na | 901 | 0.555 | 0.0201 | No |
| 8 | IL11RA | na | 1136 | 0.473 | 0.0088 | No |
| 9 | CCND2 | na | 1706 | 0.330 | -0.0330 | No |
| 10 | IL9R | na | 2743 | 0.158 | -0.1171 | No |
| 11 | SOCS4 | na | 2946 | 0.133 | -0.1317 | No |
| 12 | TYK2 | na | 3144 | 0.108 | -0.1463 | No |
| 13 | IL6R | na | 3564 | 0.056 | -0.1805 | No |
| 14 | IL22RA2 | na | 3786 | 0.030 | -0.1985 | No |
| 15 | CRLF2 | na | 4344 | -0.035 | -0.2446 | No |
| 16 | OSMR | na | 4598 | -0.060 | -0.2647 | No |
| 17 | CNTF | na | 4893 | -0.092 | -0.2878 | No |
| 18 | PRLR | na | 4924 | -0.095 | -0.2886 | No |
| 19 | IL20RA | na | 5127 | -0.115 | -0.3035 | No |
| 20 | CREBBP | na | 5240 | -0.125 | -0.3107 | No |
| 21 | IL15 | na | 5256 | -0.127 | -0.3097 | No |
| 22 | STAT5A | na | 5791 | -0.178 | -0.3513 | No |
| 23 | STAT6 | na | 5959 | -0.197 | -0.3618 | No |
| 24 | LEPR | na | 6235 | -0.228 | -0.3808 | No |
| 25 | IL12RB1 | na | 6273 | -0.232 | -0.3798 | No |
| 26 | IFNAR2 | na | 6336 | -0.238 | -0.3808 | No |
| 27 | STAM2 | na | 6431 | -0.247 | -0.3843 | No |
| 28 | PIM1 | na | 6519 | -0.256 | -0.3871 | No |
| 29 | JAK3 | na | 6534 | -0.258 | -0.3837 | No |
| 30 | IL4R | na | 6643 | -0.269 | -0.3880 | No |
| 31 | GRB2 | na | 6686 | -0.274 | -0.3866 | No |
| 32 | SOCS5 | na | 6883 | -0.295 | -0.3978 | No |
| 33 | MYC | na | 6951 | -0.302 | -0.3981 | No |
| 34 | AKT3 | na | 7033 | -0.312 | -0.3993 | No |
| 35 | CCND3 | na | 7194 | -0.329 | -0.4069 | No |
| 36 | GHR | na | 7374 | -0.349 | -0.4158 | No |
| 37 | PIAS4 | na | 7394 | -0.351 | -0.4111 | No |
| 38 | IFNGR2 | na | 7611 | -0.373 | -0.4226 | No |
| 39 | IL6ST | na | 7807 | -0.397 | -0.4319 | No |
| 40 | EP300 | na | 7828 | -0.398 | -0.4265 | No |
| 41 | CNTFR | na | 8279 | -0.455 | -0.4562 | No |
| 42 | AKT1 | na | 8399 | -0.472 | -0.4578 | Yes |
| 43 | PIK3CG | na | 8478 | -0.481 | -0.4558 | Yes |
| 44 | SPRY1 | na | 8482 | -0.481 | -0.4475 | Yes |
| 45 | STAT4 | na | 8576 | -0.496 | -0.4465 | Yes |
| 46 | SPRY2 | na | 8604 | -0.500 | -0.4399 | Yes |
| 47 | IL7 | na | 8686 | -0.511 | -0.4377 | Yes |
| 48 | SOS1 | na | 8869 | -0.533 | -0.4435 | Yes |
| 49 | IL2RG | na | 8882 | -0.534 | -0.4350 | Yes |
| 50 | CSF2RA | na | 8987 | -0.547 | -0.4340 | Yes |
| 51 | CSF3R | na | 9044 | -0.555 | -0.4289 | Yes |
| 52 | IL15RA | na | 9275 | -0.595 | -0.4376 | Yes |
| 53 | SOCS2 | na | 9342 | -0.605 | -0.4324 | Yes |
| 54 | IFNAR1 | na | 9530 | -0.636 | -0.4368 | Yes |
| 55 | PIK3R2 | na | 9597 | -0.645 | -0.4309 | Yes |
| 56 | PTPN6 | na | 9763 | -0.668 | -0.4329 | Yes |
| 57 | CSF2RB | na | 9787 | -0.672 | -0.4229 | Yes |
| 58 | PIK3CB | na | 10335 | -0.777 | -0.4550 | Yes |
| 59 | IL21R | na | 10343 | -0.779 | -0.4418 | Yes |
| 60 | CCND1 | na | 10432 | -0.796 | -0.4350 | Yes |
| 61 | LIFR | na | 10540 | -0.818 | -0.4295 | Yes |
| 62 | PTPN11 | na | 10560 | -0.822 | -0.4165 | Yes |
| 63 | IL10RB | na | 10676 | -0.848 | -0.4111 | Yes |
| 64 | PIK3CD | na | 10707 | -0.855 | -0.3984 | Yes |
| 65 | IL2RA | na | 10774 | -0.872 | -0.3885 | Yes |
| 66 | PIK3CA | na | 10920 | -0.912 | -0.3845 | Yes |
| 67 | PIK3R5 | na | 10941 | -0.918 | -0.3698 | Yes |
| 68 | CBLB | na | 10996 | -0.935 | -0.3578 | Yes |
| 69 | PIK3R3 | na | 11045 | -0.949 | -0.3450 | Yes |
| 70 | STAT3 | na | 11052 | -0.950 | -0.3286 | Yes |
| 71 | IL10RA | na | 11062 | -0.955 | -0.3124 | Yes |
| 72 | CISH | na | 11064 | -0.955 | -0.2956 | Yes |
| 73 | PIAS2 | na | 11080 | -0.959 | -0.2798 | Yes |
| 74 | JAK2 | na | 11123 | -0.971 | -0.2661 | Yes |
| 75 | SOCS3 | na | 11181 | -0.994 | -0.2532 | Yes |
| 76 | JAK1 | na | 11193 | -0.997 | -0.2365 | Yes |
| 77 | MPL | na | 11201 | -1.000 | -0.2193 | Yes |
| 78 | PIK3R1 | na | 11265 | -1.023 | -0.2064 | Yes |
| 79 | SPRED2 | na | 11306 | -1.040 | -0.1913 | Yes |
| 80 | SOS2 | na | 11362 | -1.063 | -0.1771 | Yes |
| 81 | PIAS1 | na | 11577 | -1.157 | -0.1745 | Yes |
| 82 | IL7R | na | 11582 | -1.161 | -0.1543 | Yes |
| 83 | SPRY3 | na | 11641 | -1.197 | -0.1379 | Yes |
| 84 | STAM | na | 11654 | -1.202 | -0.1176 | Yes |
| 85 | IL13RA1 | na | 11716 | -1.255 | -0.1004 | Yes |
| 86 | IL12RB2 | na | 11728 | -1.267 | -0.0789 | Yes |
| 87 | IL22 | na | 11774 | -1.308 | -0.0594 | Yes |
| 88 | CBL | na | 11873 | -1.446 | -0.0420 | Yes |
| 89 | IL2RB | na | 11878 | -1.457 | -0.0165 | Yes |
| 90 | IFNGR1 | na | 11917 | -1.547 | 0.0078 | Yes |
Table: GSEA details [plain text format]

  

Fig 2: KEGG\_JAK\_STAT\_SIGNALING\_PATHWAY      
 Blue-Pink O' Gram in the Space of the Analyzed GeneSet

  

Fig 3: KEGG\_JAK\_STAT\_SIGNALING\_PATHWAY: Random ES distribution      
 Gene set null distribution of ES for **KEGG\_JAK\_STAT\_SIGNALING\_PATHWAY**
